# Supplementary material for: Green inspired synthesis of zinc oxide nanoparticles using Silybum marianum (milk thistle) extract and evaluation of their potential pesticidal and phytopathogens activities
Source: PeerJ. 2023 Aug 14;11:e15743. doi: 10.7717/peerj.15743 (PMC10434149; doi:10.7717/peerj.15743)
Supplement: Supplemental Information 1 [file peerj-11-15743-s001.docx]

**Green Inspired Synthesis of Zinc Oxide Nanoparticles using *Silybum marianum* (milk thistle) extract and Evaluation of their Potential Pesticidal and Phytopathogens Activities**

Nazish Jahan^1^, Kousar Rasheed^1^, Khalil-Ur-Rahman^2^, Abu Hazafa^3,*^, Amna Saleem^1^, Saud Alamri^4^, Muhammad Omer Iqbal^5^, Md Atikur Rahman^6^

Affiliations

*^1^ Department of Chemistry, Faculty of Sciences, University of Agriculture Faisalabad, 38040, Pakistan*

*^2^ Department of Biochemistry, Riphah International University, Faisalabad, Pakistan*

*^3^ Department of Biochemistry, Faculty of Sciences, University of Agriculture Faisalabad, 38040, Pakistan*

*^4^ Department of Botany and Microbiology, College of Science, King Saud University, Riyadh-11451, Saudi Arabia*

*^5^ Key Laboratory of Marine Drugs, the Ministry of Education, School of Medicine and Pharmacy, Ocean University of China, Qingdao 266003, China*

*^6^ Grassland & Forages Division, National Institute of Animal Science, Rural Development Administration, Cheonan, 330-801, Republic of Korea*

*** Corresponding author:**

Email: [ahazafa@unisa.it](mailto:ahazafa@unisa.it) or Abu.9093270@talmeez.pk (A. Hazafa)

**List of Figures**

**
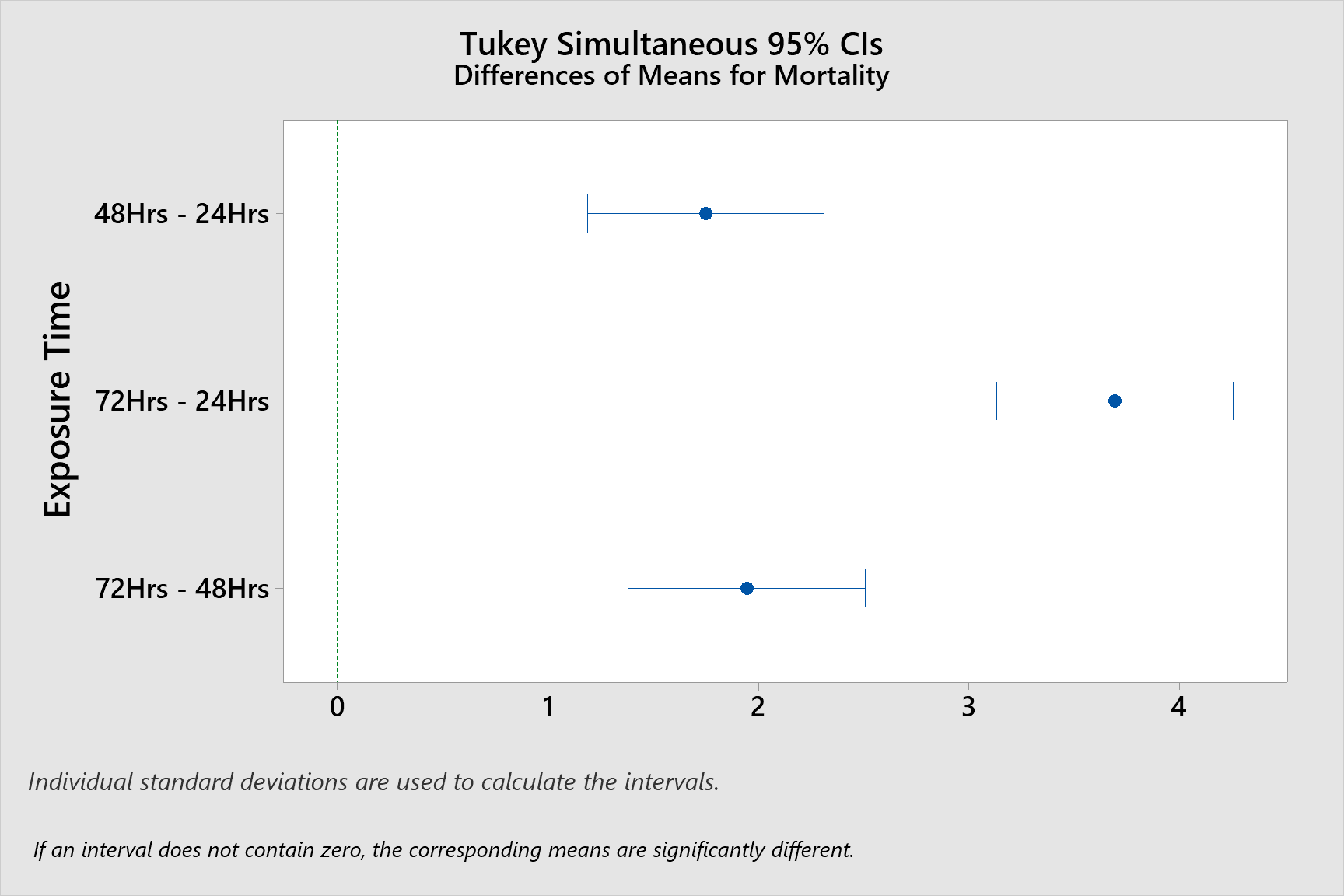
**

**Fig. 1.** Tukey pairwise comparisons of pesticidal results of *Sitophilus oryzae* using the Tukey Method and 95% CIs.

**
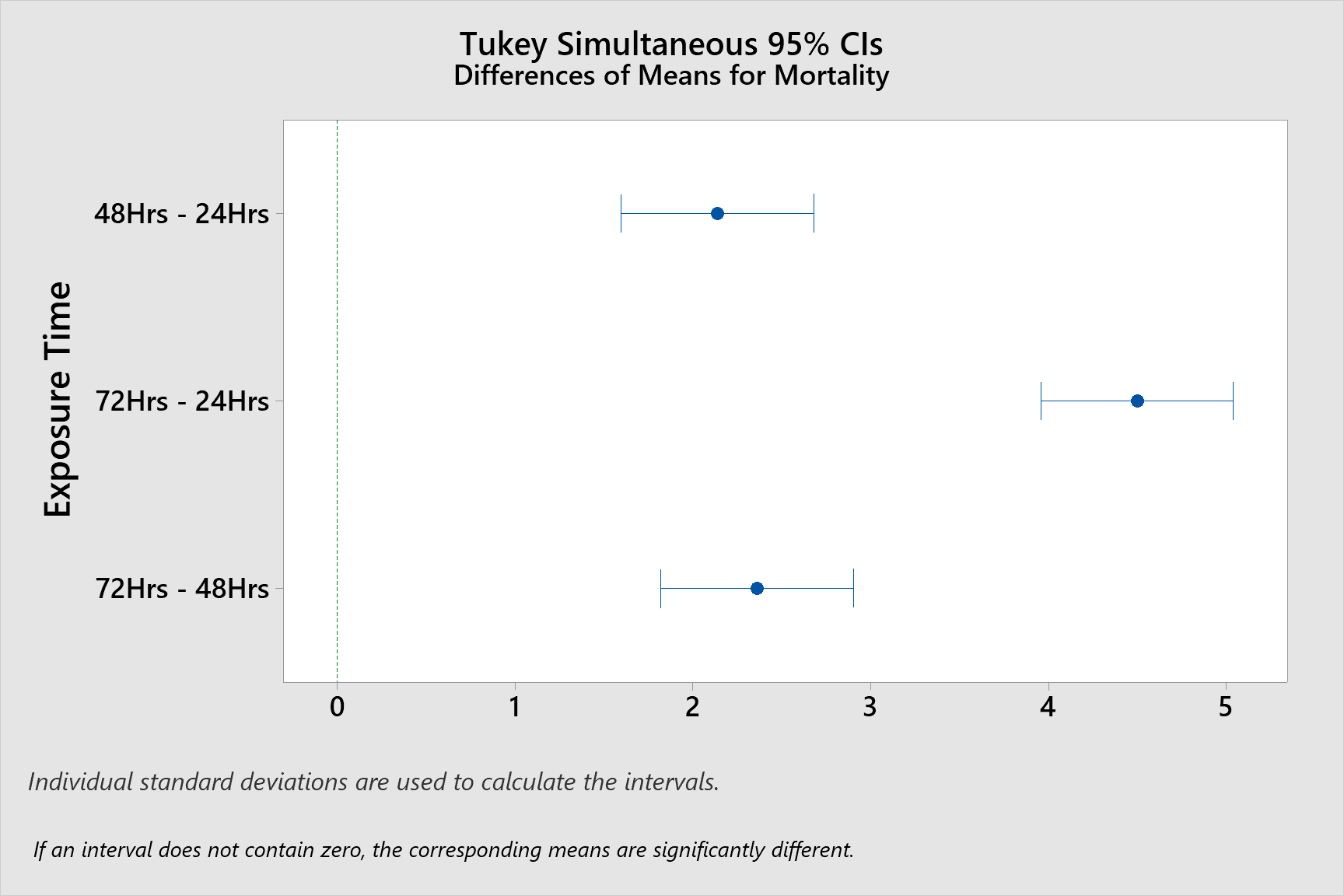
**

**Fig. 2.** Tukey pairwise comparisons of pesticidal results of *Tribolium castaneum* using the Tukey Method and 95% CIs.


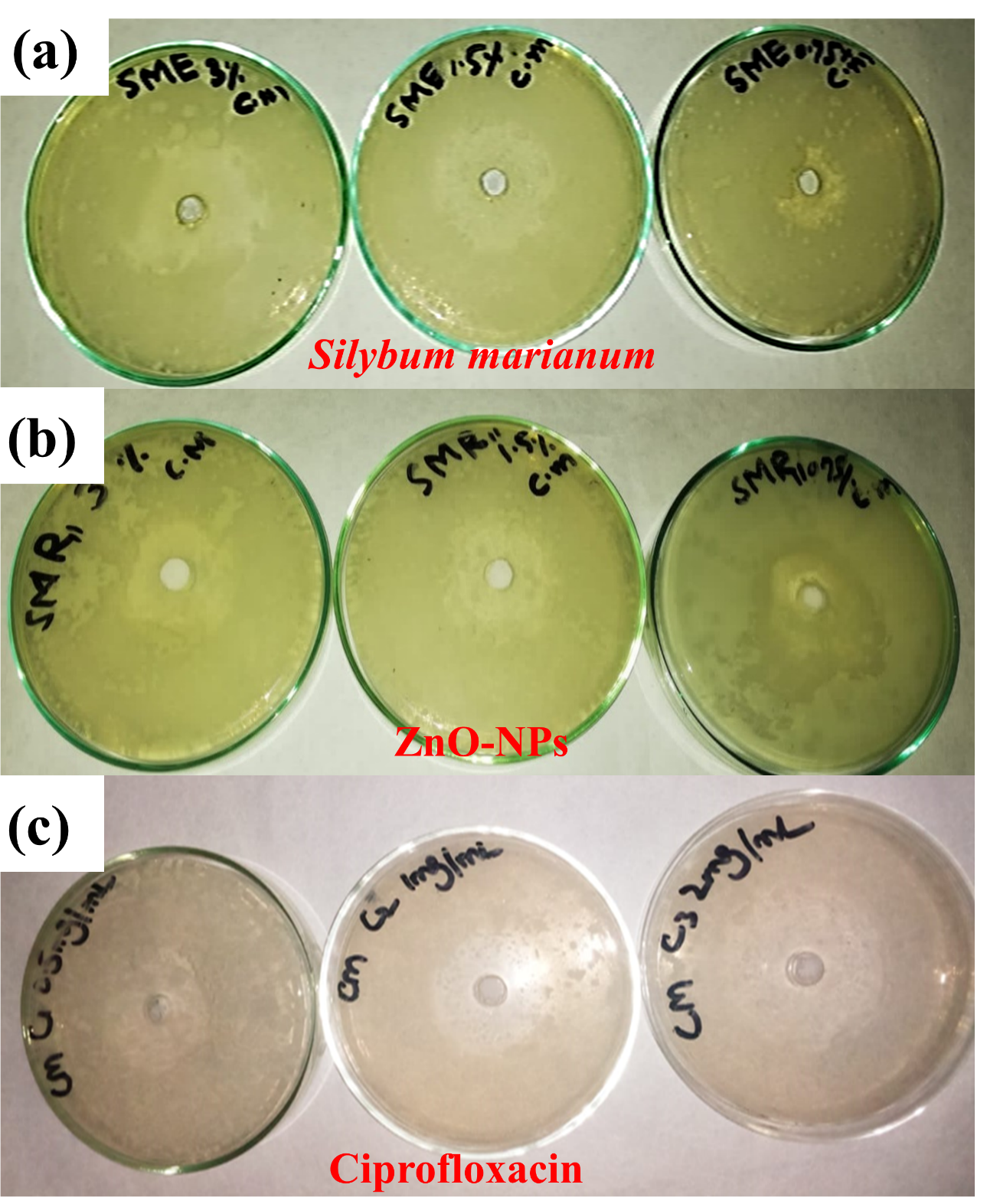


**Fig. 3.** Antibacterial activity of (a) *Silybum marianum* seed extract, (b) ZnO-NPs, and (c) ciprofloxacin against *Clavibacter michiganensis* at different concentrations.


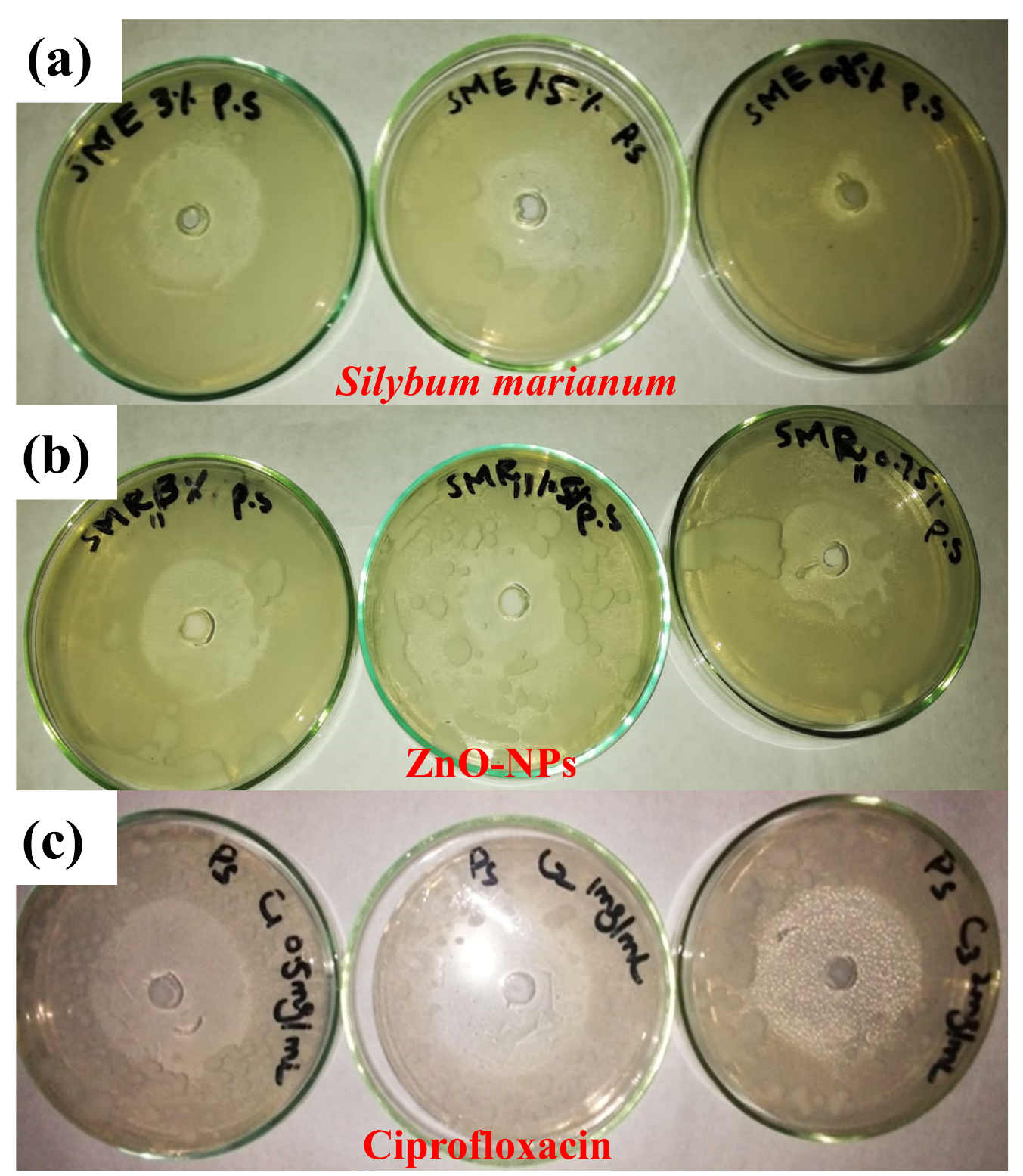


**Fig. 4**. Antibacterial activity of (a) *Silybum marianum* seed extract, (b) ZnO-NPs, and (c) ciprofloxacin against *Pseudomonas syringae* at different concentrations.


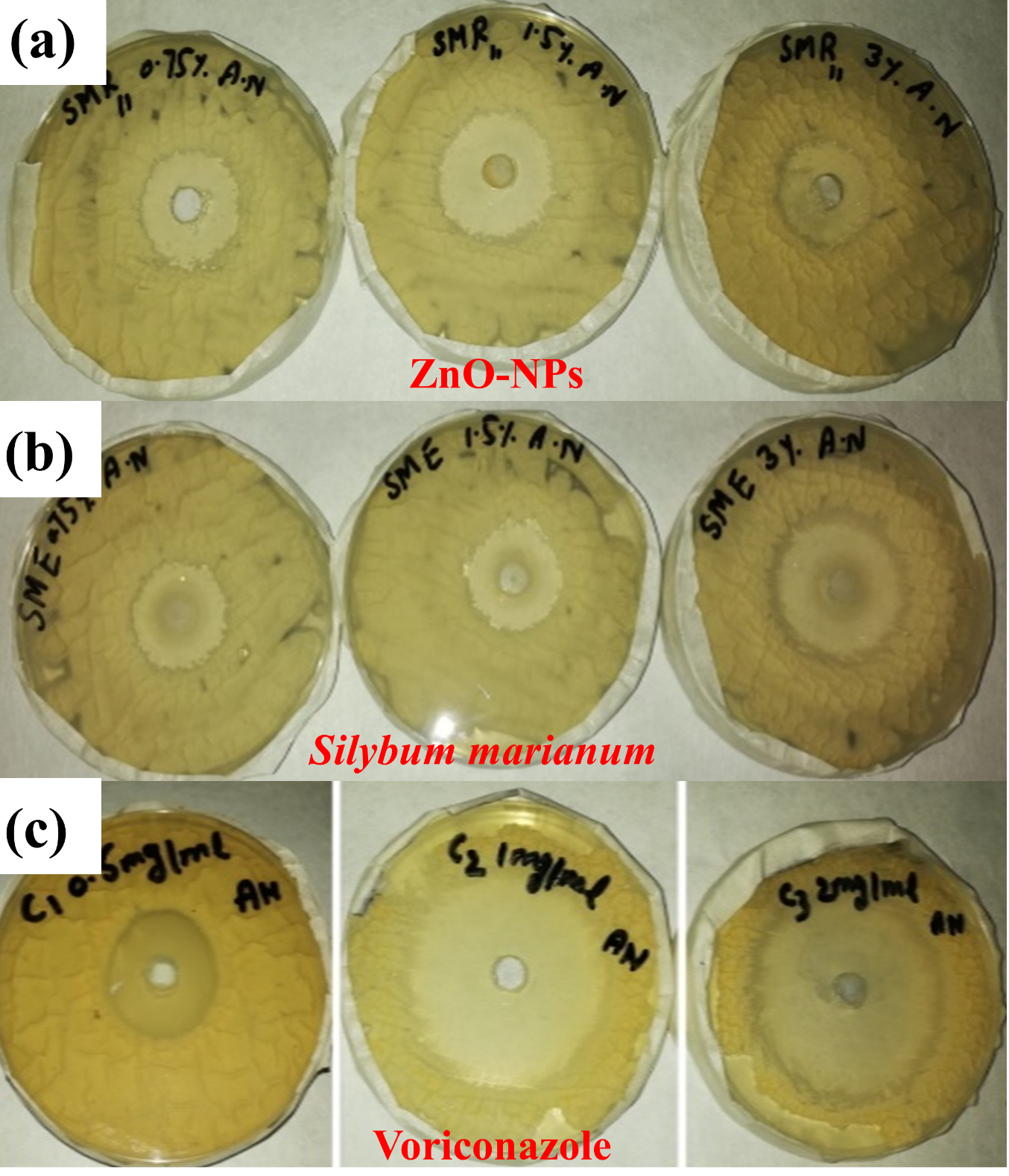


**Fig. 5.** Antifungal activity of (a) ZnO-NPs, (b) *Silybum marianum* seed extract, and (c) voriconazole against *Aspergillus niger*.


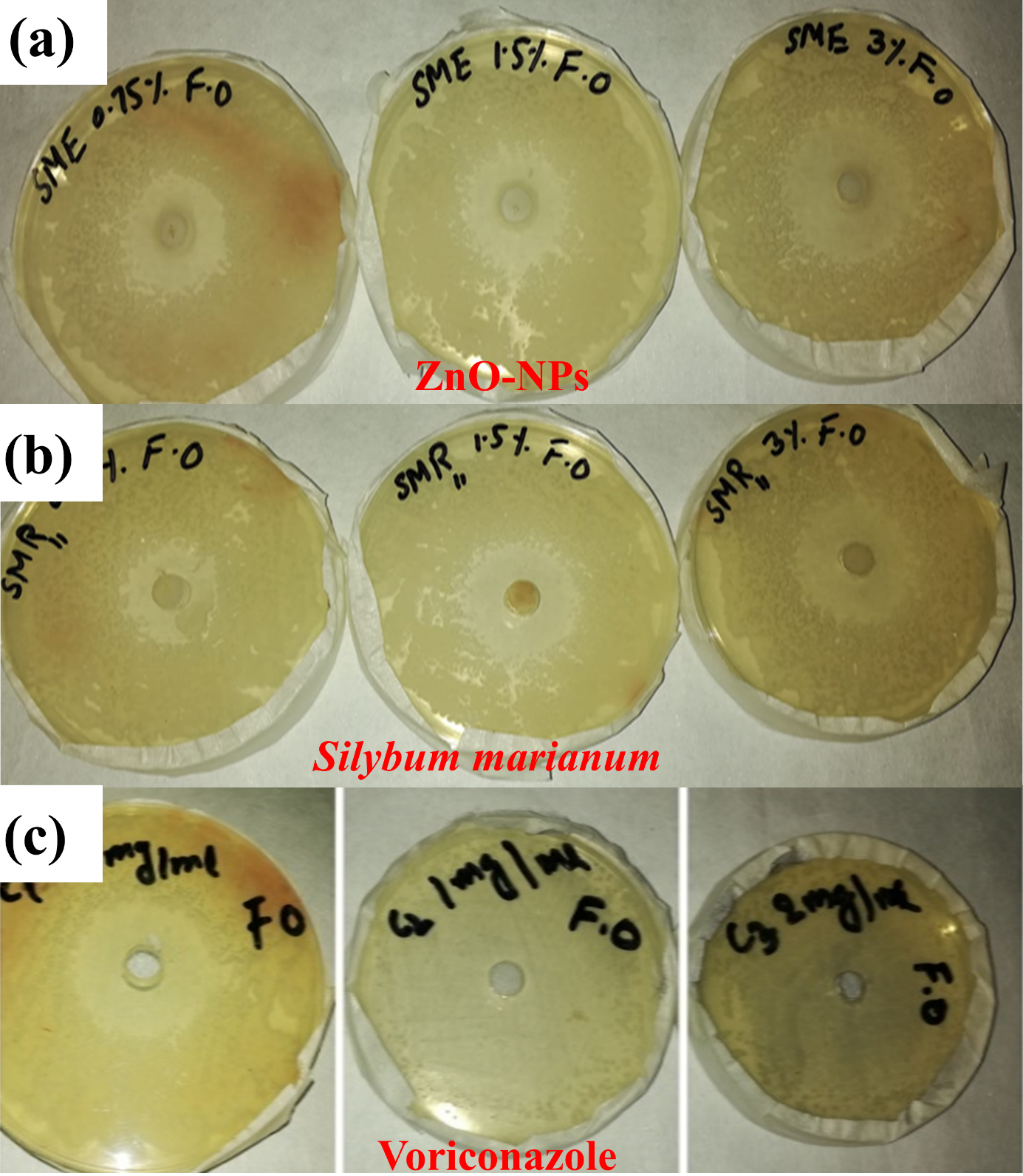


**Fig. 6.** Antifungal activity of (a) ZnO-NPs, (b) *Silybum marianum* seed extract, and (c) voriconazole *Fusarium oxysporum*.

**List of Tables**

**Table 1**. Results of analysis of variance of *Sitophilus oryzae* among inhibition zone versus sources and treatments.

| **Source** | **DF** | **Adj SS** | **Adj MS** | **F-Value** | **P-Value** |
| --- | --- | --- | --- | --- | --- |
| Treatments | 11 | 2328.52 | 211.684 | 211.68 | 0.000 |
| Exposure time | 2 | 245.91 | 122.954 | 122.95 | 0.000 |
| Treatments*Exposure time | 22 | 124.76 | 5.671 | 5.67 | 0.000 |
| Error | 72 | 72.00 | 1.000 |  |  |
| Total | 107 | 2771.17 |  |  |  |

**Table 2**. Results of analysis of variance of *Sitophilus oryzae* among inhibition zone versus sources and treatments.

| **Source** | **DF** | **Adj SS** | **Adj MS** | **F-Value** | **P-Value** |
| --- | --- | --- | --- | --- | --- |
| Treatments | 11 | 1885.07 | 171.370 | 185.08 | 0.000 |
| Exposure time | 2 | 364.80 | 182.398 | 196.99 | 0.000 |
| Treatments*Exposure time | 22 | 128.98 | 5.863 | 6.33 | 0.000 |
| Error | 72 | 66.67 | 0.926 |  |  |
| Total | 107 | 2445.52 |  |  |  |

**Table 3**. Results of analysis of variance of *Clavibacter michiganensis* among inhibition zone versus sources and treatments.

| **Source** | **DF** | **Adj SS** | **Adj MS** | **F-Value** | **P-Value** |
| --- | --- | --- | --- | --- | --- |
| Sources | 2 | 1319.36 | 659.680 | 2736.00 | 0.000 |
| Treatments | 2 | 645.81 | 322.903 | 1339.23 | 0.000 |
| Sources*Treatments | 4 | 10.60 | 2.650 | 10.99 | 0.000 |
| Error | 18 | 4.34 | 0.241 |  |  |
| Total | 26 | 1980.11 |  |  |  |

**Table 4**. Tukey pairwise comparisons of antibacterial results of *Clavibacter michiganensis* using Tukey Method and 95% confidence.

| **Sources*Treatments** | **N** | **Mean** | **Grouping** | | | | | | | |
| --- | --- | --- | --- | --- | --- | --- | --- | --- | --- | --- |
| Control T3 | 3 | 33.633 | A |  |  |  |  |  |  |  |
| Control T2 | 3 | 26.833 |  | B |  |  |  |  |  |  |
| Control T1 | 3 | 19.567 |  |  | C |  |  |  |  |  |
| ZnO-NPs T3 | 3 | 17.633 |  |  |  | D |  |  |  |  |
| Plant extract T3 | 3 | 16.633 |  |  |  | D |  |  |  |  |
| ZnO-NPs T2 | 3 | 13.033 |  |  |  |  | E |  |  |  |
| Plant extract T2 | 3 | 11.533 |  |  |  |  |  | F |  |  |
| ZnO-NPs T1 | 3 | 6.567 |  |  |  |  |  |  | G |  |
| Plant extract T2 | 3 | 5.867 |  |  |  |  |  |  | G |  |

Response = Inhibition zone, Term = Sources*Treatments

**Table 5**. Results of analysis of variance of *Pseudomonas syringae* among inhibition zone versus sources and treatments.

| **Source** | **DF** | **Adj SS** | **Adj MS** | **F-Value** | **P-Value** |
| --- | --- | --- | --- | --- | --- |
| Sources | 2 | 975.65 | 487.827 | 1699.53 | 0.000 |
| Treatments | 2 | 911.17 | 455.584 | 1587.19 | 0.000 |
| Sources*Treatments | 4 | 12.27 | 3.068 | 10.69 | 0.000 |
| Error | 18 | 5.17 | 0.287 |  |  |
| Total | 26 | 1904.26 |  |  |  |

**Table 6**. Tukey pairwise comparisons of antibacterial results of *Pseudomonas syringae* using Tukey Method and 95% confidence.

| **Sources*Treatments** | **N** | **Mean** | **Grouping** | | | | | | | |
| --- | --- | --- | --- | --- | --- | --- | --- | --- | --- | --- |
| Control T3 | 3 | 35.467 | A |  |  |  |  |  |  |  |
| Control T2 | 3 | 28.833 |  | B |  |  |  |  |  |  |
| ZnO-NPs T3 | 3 | 24.500 |  |  | C |  |  |  |  |  |
| Control T1 | 3 | 20.366 |  |  |  | D |  |  |  |  |
| Plant extract T3 | 3 | 19.766 |  |  |  | D | E |  |  |  |
| ZnO-NPs T2 | 3 | 18.500 |  |  |  |  | E |  |  |  |
| Plant extract T2 | 3 | 14.933 |  |  |  |  |  | F |  |  |
| ZnO-NPs T1 | 3 | 9.033 |  |  |  |  |  |  | G |  |
| Plant extract T2 | 3 | 7.866 |  |  |  |  |  |  | G |  |

Response = Inhibition zone, Term = Sources*Treatments

**Table 7**. Results of analysis of variance of *Aspergillus niger* among inhibition zone versus sources and treatments.

| **Source** | **DF** | **Adj SS** | **Adj MS** | **F-Value** | **P-Value** |
| --- | --- | --- | --- | --- | --- |
| Sources | 2 | 850.94 | 425.470 | 1298.04 | 0.000 |
| Treatments | 2 | 602.42 | 301.210 | 918.95 | 0.000 |
| Sources*Treatments | 4 | 85.29 | 21.322 | 65.05 | 0.000 |
| Error | 18 | 5.90 | 0.328 |  |  |
| Total | 26 | 1544.55 |  |  |  |

**Table 8.** Tukey pairwise comparisons of antifungal results of *Aspergillus niger* using Tukey Method and 95% confidence.

| **Sources*Treatments** | **N** | **Mean** | **Grouping** | | | | | | | |
| --- | --- | --- | --- | --- | --- | --- | --- | --- | --- | --- |
| Control T3 | 3 | 30.133 | A |  |  |  |  |  |  |  |
| Control T2 | 3 | 27.333 |  | B |  |  |  |  |  |  |
| ZnO-NPs T3 | 3 | 18.533 |  |  | C |  |  |  |  |  |
| Plant extract T3 | 3 | 17.400 |  |  | C |  |  |  |  |  |
| Control T1 | 3 | 15.200 |  |  |  | D |  |  |  |  |
| ZnO-NPs T2 | 3 | 11.500 |  |  |  |  | E |  |  |  |
| Plant extract T2 | 3 | 10.633 |  |  |  |  | E | F |  |  |
| ZnO-NPs T1 | 3 | 9.133 |  |  |  |  |  | F |  |  |
| Plant extract T2 | 3 | 7.033 |  |  |  |  |  |  | G |  |

Response = Inhibition zone, Term = Sources*Treatments

**Table 9**. Results of analysis of variance of *Fusarium oxysporum* among inhibition zone versus sources and treatments.

| **Source** | **DF** | **Adj SS** | **Adj MS** | **F-Value** | **P-Value** |
| --- | --- | --- | --- | --- | --- |
| Sources | 2 | 910.14 | 455.069 | 1692.41 | 0.000 |
| Treatments | 2 | 555.88 | 277.939 | 1033.66 | 0.000 |
| Sources*Treatments | 4 | 135.76 | 33.941 | 126.23 | 0.000 |
| Error | 18 | 4.84 | 0.269 |  |  |
| Total | 26 | 1606.62 |  |  |  |

**Table 10**. Tukey pairwise comparisons of antifungal results of *Fusarium oxysporum* using Tukey Method and 95% confidence.

| **Sources*Treatments** | **N** | **Mean** | **Grouping** | | | | | | | |
| --- | --- | --- | --- | --- | --- | --- | --- | --- | --- | --- |
| Control T3 | 3 | 35.000 | A |  |  |  |  |  |  |  |
| Control T2 | 3 | 30.833 |  | B |  |  |  |  |  |  |
| ZnO-NPs T3 | 3 | 20.700 |  |  | C |  |  |  |  |  |
| Plant extract T3 | 3 | 18.533 |  |  |  | D |  |  |  |  |
| Control T1 | 3 | 17.400 |  |  |  | D |  |  |  |  |
| ZnO-NPs T2 | 3 | 15.500 |  |  |  |  | E |  |  |  |
| Plant extract T2 | 3 | 14.566 |  |  |  |  | E |  |  |  |
| ZnO-NPs T1 | 3 | 12.600 |  |  |  |  |  | F |  |  |
| Plant extract T2 | 3 | 11.100 |  |  |  |  |  |  | G |  |

Response = Inhibition zone, Term = Sources*Treatments
