# Supplementary material for: Green inspired synthesis of zinc oxide nanoparticles using Silybum marianum (milk thistle) extract and evaluation of their potential pesticidal and phytopathogens activities
Source: PeerJ. 2023 Aug 14;11:e15743. doi: 10.7717/peerj.15743 (PMC10434149; doi:10.7717/peerj.15743)
Supplement: Supplemental Information 2 [file peerj-11-15743-s002.pdf]

Pesticidal and antimicrobial activities

Pesticidal activity against Sitophilus oryzae (%)

		Plant extract	ZnO-NPs	Control
T1 (0.75 %)	24H	3	9	37.7
	48H	5	25	64.4
	72H	7	34	91.1
T2 (1.5%)	24H	5	16	55.5
	48H	7	29	80
	72H	12	43	95.5
T3 (3%)	24H	9	20	75.55
	48H	16	36	86.6
	72H	23	58	100
T4 (6%)	24H	16	23	89.5
	48H	18	38	93.2
	72H	25	74	95.5

Pesticidal activity against Tribolium castaneum (%)

		Plant extract	ZnO-NPs	Control
T1 (0.75 %)	24H	5	9	33.3
	48H	7	27	62.2
	72H	9	43	88.8
T2 (1.5%)	24H	9	18	51
	48H	16	34	77.7
	72H	23	56	93.33
T3 (3%)	24H	12	20	71.1
	48H	20	45	84.4
	72H	28	69	97.7
T4 (6%)	24H	18	25	89.5
	48H	27	45	93.2
	72H	43	78	95.5

Antibacterial activity of Silybum marianum against Clavibacter michiganensis (mm)			
	Plant extract	ZnO-NPs	Control
T1 (0.5%)	6	8	19.8
T2 (1.5%)	12	14	26.8
T3 (3%)	17	18	33.6

Antibacterial activity of Silybum marianum against Pseudomonas syringae (mm)			
	Plant extract	ZnO-NPs	Control
T1 (0.5%)	8	10	20.3
T2 (1.5%)	15	19	28.8
T3 (3%)	20	25	35.4

Antifungal activity gainst Aspergillums niger (mm)			
	Plant extract	ZnO-NPs	Control
T1 (0.5%)	7	10	15.2
T2 (1.5%)	11	12	27.3
T3 (3%)	18	19	30.1

Antifungal activity gainst Fusarium oxysporum (mm)			
	Plant extract	ZnO-NPs	Control
T1 (0.5%)	12	13	17.4
T2 (1.5%)	15	16	30.8
T3 (3%)	18	21	35
